# Disorganization of white matter architecture in major depressive disorder: a meta-analysis of diffusion tensor imaging with tract-based spatial statistics

**DOI:** 10.1038/srep21825

**Published:** 2016-02-24

**Authors:** Guangxiang Chen, Xinyu Hu, Lei Li, Xiaoqi Huang, Su Lui, Weihong Kuang, Hua Ai, Feng Bi, Zhongwei Gu, Qiyong Gong

**Affiliations:** 1 Huaxi MR Research Center (HMRRC), Department of Radiology, West China Hospital, Sichuan University, Chengdu 610041, Sichuan, China; 2 Department of Radiology, The Affiliated Hospital of Luzhou Medical College, Luzhou 646000, Sichuan, China; 3 Department of Psychiatry, State Key Lab of Biotherapy, West China Hospital, Sichuan University, Chengdu 610041, Sichuan, China; 4 National Engineering Research Center for Biomaterials, Sichuan University, Chengdu 610064, Sichuan, China; 5 Department of Oncology, State Key Lab of Biotherapy, West China Hospital, Sichuan University, Chengdu 610041, Sichuan, China; 6 Department of Psychology, School of Public Administration, Sichuan University, Chengdu 610041, Sichuan, China

## Abstract

White matter (WM) abnormalities have long been suspected in major depressive disorder (MDD). Tract-based spatial statistics (TBSS) studies have detected abnormalities in fractional anisotropy (FA) in MDD, but the available evidence has been inconsistent. We performed a quantitative meta-analysis of TBSS studies contrasting MDD patients with healthy control subjects (HCS). A total of 17 studies with 18 datasets that included 641 MDD patients and 581 HCS were identified. Anisotropic effect size-signed differential mapping (AES-SDM) meta-analysis was performed to assess FA alterations in MDD patients compared to HCS. FA reductions were identified in the genu of the corpus callosum (CC) extending to the body of the CC and left anterior limb of the internal capsule (ALIC) in MDD patients relative to HCS. Descriptive analysis of quartiles, sensitivity analysis and subgroup analysis further confirmed these findings. Meta-regression analysis revealed that individuals with more severe MDD were significantly more likely to have FA reductions in the genu of the CC. This study provides a thorough profile of WM abnormalities in MDD and evidence that interhemispheric connections and frontal-striatal-thalamic pathways are the most convergent circuits affected in MDD.

Major depressive disorder (MDD) is a common chronically debilitating psychiatric disorder with an estimated prevalence of 13% to 16% in the general population^1^,^2^. MDD is characterized by the profound dysregulation of mood as well as additional abnormalities including cognitive dysfunction, insomnia, fatigue and appetite disturbance^3^. Despite psychopharmacologic and psychotherapeutic treatments, MDD remains a costly mental health illness in terms of total health care expenditures and lost productivity^4^. Consequently, a greater understanding of the neural correlates underlying MDD is of great significance to identify biologically based targets to improve the specificity and efficacy of diagnostic and treatment strategies for MDD.

Over the last few decades, modern imaging techniques have greatly increased our knowledge of MDD, particularly its neural bases. Previous studies of structural and functional magnetic resonance imaging (MRI) have reported various grey matter (GM) abnormalities in MDD patients, including abnormalities in the prefrontal cortex^5^,^6^,^7^, anterior cingulate cortex^8^,^9^, hippocampus^10^,^11^ and thalamus^12^,^13^. These observations suggest that a dysfunctional prefrontal-limbic circuit instead of a deficit in distinct regions plays an important role in the pathophysiology of MDD. As the infrastructure connecting those cortical and subcortical regions and the basis for structure connectivity, white matter (WM) warrants more exploration.

In contrast to conventional T1-weighted structural images of WM in the brain, diffusion tensor imaging (DTI), a noninvasive magnetic resonance method based on the diffusion characteristics of water, can be used to quantify the fibre orientation and integrity of WM pathways within neural networks^14^,^15^. One commonly used parameter for measuring WM integrity is fractional anisotropy (FA), an invariant property of DTI that reflects a nonspherical diffusion tensor with a preferential orientation^16^,^17^,^18^. FA can be measured using two techniques: the region of interest (ROI) approach, which manually preselects limited and potentially biased parts of the brain for analysis, and whole-brain study including voxel-based analysis (VBA) and tract-based spatial statistics (TBSS), which generally report the three-dimensional coordinates for which there are maximal FA differences in patients compared with healthy control subjects (HCS).

FA reduction is related to depression severity and illness duration in MDD patients^19^, which indicates that DTI may be of clinical value in measuring and tracking disability in MDD. However, many studies have reported inconsistent and controversial results due to small and heterogeneous samples and substantial methodological differences between studies. For example, several studies have reported FA reductions in the right frontal WM, left lateral occipital WM, left superior longitudinal fasciculus, and left anterior limb of the internal capsule (ALIC)^20^,^21^,^22^,^23^. However, another study with a large sample size observed no significant differences in FA between MDD patients and HCS^24^. Thus, there has been increasing interest in meta-analysis to identify consistent results for DTI studies in MDD and provide more insight into the neural-anatomical basis for structural connections in this disorder.

However, a major shortcoming of published voxel-wise meta-analyses of DTI studies of MDD is the use of both VBA and TBSS studies^25^,^26^,^27^. VBA is relatively straightforward and involves the spatial normalization of high-resolution images from all of the subjects in the study to the same stereotactic space^28^. TBSS is a statistical method in which each subject’s FA data are projected onto the mean FA skeleton such that each skeleton voxel takes the FA value from the local centre of the nearest relevant tract, thus alleviating the misalignment problems that can arise in regular VBA studies due to anatomical difference between groups^29^. Furthermore, Wise *et al.* published a meta-analysis integrating both TBSS and VBA studies to investigate the structural disconnectivity in MDD and they demonstrated that TBSS might be a more sensitive technique for the detection of WM abnormalities and provide a more accurate estimate compared with VBA^27^. However, in the study conducted by Wise *et al.*^27^, only 10 DTI studies with TBSS were included, and other confounding factors, such as medication status, were not considered. Furthermore, they did not find the association between the symptom severity evaluated by Hamilton Depression Rating Scale (HAMD) and neuroimaging alterations because only 7 TBSS datasets reporting HAMD scores were included in Wise's research while according to Radua *et al.*^30^, the meta-regression analysis is invalid if data is available for fewer than 9 datasets. Since more original TBSS studies regarding MDD have been published in recent years, it is worthwhile to conduct an updated tract-based spatial meta-analysis to explore the WM microstructure abnormalities and investigate the effects of symptom severity and other clinical profiles on regional WM alterations.

Therefore, the goals of this study were threefold: first, we conducted an updated quantitative summary of 17 TBSS studies (14 TBSS datasets reported HAMD scores) concerning FA abnormalities in MDD using anisotropic effect size-signed differential mapping (AES-SDM), a newly developed meta-analytic technique with the potential to quantify the reproducibility of neuroimaging findings and to generate insights that are difficult to obtain from an individual study; second, we performed subgroup meta-analyses to compare first-episode, treatment-naive/medication-free MDD patients with HCS to avoid the potential confounding effects of medication; third, we used a meta-regression method to examine the potentially moderating effects of symptom severity and other relevant variables on the reported WM abnormalities.

## Results

### Included studies and sample characteristics

**Figure 1:**
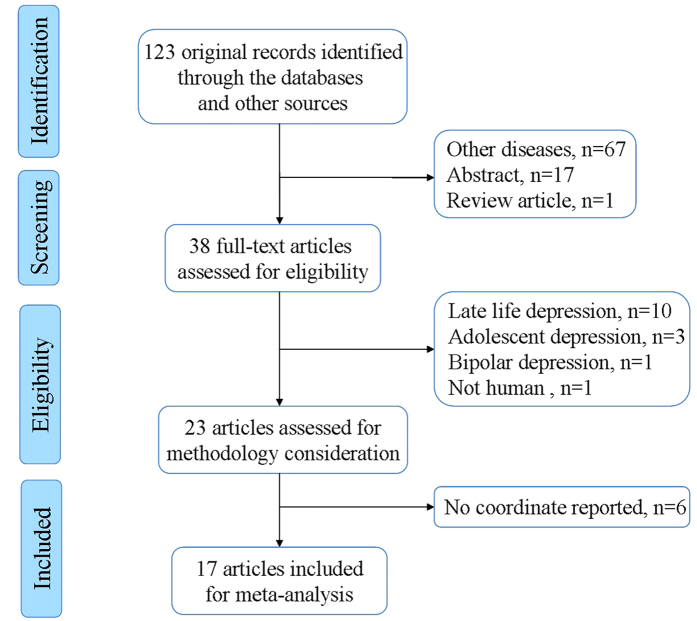
Flow diagram for the identification and exclusion of studies.

The search strategy yielded a total of 123 studies, of which 17 TBSS studies^20^,^21^,^22^,^23^,^24^,^31^,^32^,^33^,^34^,^35^,^36^,^37^,^38^,^39^,^40^,^41^,^42^ with 18 datasets met the inclusion criteria. The included studies reported FA alterations in WM in 641 individuals with MDD (255 male and 386 female; mean age 37.4 years) relative to 581 HCS (263 male and 318 female; mean age 33.4 years). In one study^37^, suicide attempters and non-attempters with MDD were compared with the same healthy participants. Thus, we treated this study as two unique and independent datasets in the meta-analysis. The flow diagram of the identification and exclusion of studies is presented in Fig. 1. Table 1 summarizes the characteristics of these studies included in the meta-analysis.

### Regional difference in FA in all included studies

**Figure 2:**
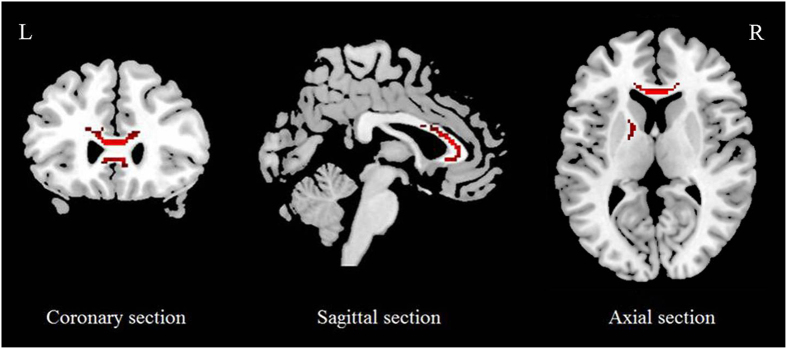
Regional FA reductions in the genu and body of the CC as well as the left ALIC in MDD patients compared with HCS. Significant clusters are overlaid on an MRIcron template for Windows for display purposes only. Abbreviations: FA, fractional anisotropy; CC, corpus callosum; ALIC, anterior limb of internal capsule; MDD, major depressive disorder; HCS, healthy control subjects.

**Figure 3:**
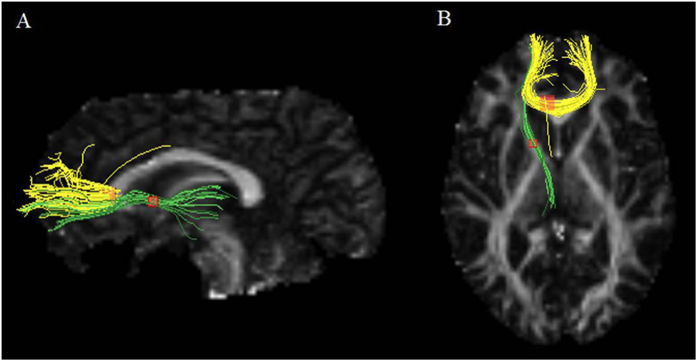
Three-dimensional images showing white matter tracts traversing two bounding boxes centred at x = −6, y = 26, z = 10 and x = −14, y = 4 and z = 8 were separately mapped with DTIquery in a single normal individual. Left image (**A**) observed from the left side of the brain, right image (**B**) observed from above. Tracts include the interhemispheric fibres running through the genu of the CC (yellow) and the anterior thalamic radiation running through the left ALIC (green). Sagittal and axial slices mapping the FA values are shown in the background for illustrative purposes. Abbreviations: CC, corpus callosum; ALIC, anterior limb of internal capsule; FA, fractional anisotropy.

Coordinates for the AES-SDM analysis were obtained from all 18 datasets representing 641 patients with MDD and 581 HCS. As shown in Fig. 2 and Table 2, patients with MDD had significant FA reductions in 2 clusters relative to HCS. The largest cluster exhibited a peak in the genu of the corpus callosum (CC) extending to the body of the CC. The main tracts passing through this region were the interhemispheric fibres connecting the prefrontal and orbitofrontal cortices, shown as yellow tracts in Fig. 3. The other cluster exhibited FA reduction in the left ALIC. The main tracts passing through this region were the anterior thalamic radiation connecting the medial dorsal thalamic nuclei with the prefrontal cortices, shown as the green tracts in Fig. 3. No FA increases in any of the regions were reported in the analysis. The results from Egger’s test revealed no strong evidence for publication bias in the genu of the CC (P = 0.198) and left ALIC (P = 0.123).

### Jack-knife sensitivity analysis

As shown in Table 3, whole-brain jack-knife sensitivity analysis of the pooled meta-analysis indicated that the FA reduction in patients with MDD in the genu of the CC was highly replicable; this finding was preserved throughout all 18 combinations of the datasets. The FA reduction in the left ALIC remained significant in all but three combinations of the datasets.

### Descriptive analysis of quartiles

The descriptive analysis of quartiles demonstrated that the FA reduction in the genu of the CC was detected in the median analysis, indicating that at least 50% of the included datasets detected a significant FA reduction in this region. The left ALIC was not detected in this analysis, indicating that less than 25% of the datasets detected significant FA reduction in this region (Table 3).

### Subgroup analysis of first-episode, treatment-naive and medication-free datasets

The subgroup analysis revealed that the above findings remained largely unchanged when only the 7 datasets with first-episode, treatment-naive MDD or only the 14 datasets with medication-free MDD were analysed. However, the above-mentioned analysis revealed no significant differences in the FA in the genu of the CC between patients and HCS when only the 7 datasets with first-episode, treatment-naive MDD were analysed (Table 3).

### Meta-regression analysis

**Figure 4:**
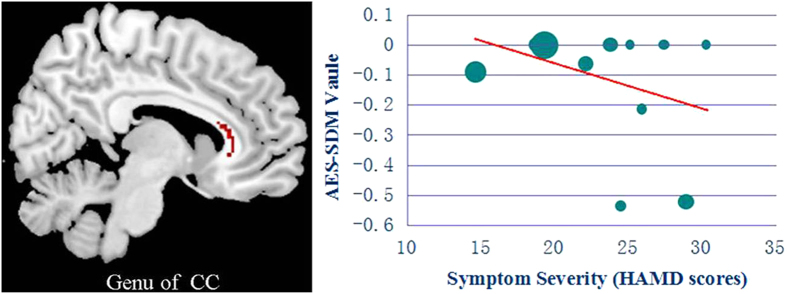
Result of the meta-regression analysis demonstrating that the symptom severity (HAMD scores) of MDD patients is negatively correlated with FA in the genu of the CC. In the graphs, AES-SDM values needed to create this plot were extracted from the peak of maximum slope significance, and each study is represented as a dot; the dot size reflects the sample size. The regression line (meta-regression signed differential mapping slope) is presented as a straight line. Abbreviations: FA, fractional anisotropy; CC, corpus callosum; MDD, major depressive disorder; HAMD, Hamilton Depression Rating Scale; AES-SDM, anisotropic effect size-signed differential mapping.

The symptom severity evaluated by HAMD scores was negatively associated with FA reduction in the genu of the CC (Montreal Neurological Institute coordinate: x = 12, y = 30, z = 8; AES-SDM value = −0.102, p = 0.00012; 129 voxels), as shown in Fig. 4. The mean age, illness duration, and drug status of MDD participants were not associated with MDD-related FA reductions in the genu of the CC, at least linearly. However, there was no significant correlation between FA reduction in the left ALIC and symptom severity or other variables.

## Discussion

This study pooled the largest number of DTI studies using TBSS to date for a meta-analysis of the difference in FA between patients with MDD and HCS. The present voxel-wise meta-analysis using AES-SDM primarily revealed that patients with MDD have FA reductions in the genu of the CC extending to the body of the CC and left ALIC. The results remained largely unchanged when each dataset was discarded individually (jack-knife sensitivity analysis). Descriptive analysis of quartiles further revealed that most of the datasets detected some degree of FA reduction in the genu of the CC and that less than 25% of the datasets detected some degree of FA reduction in the left ALIC.

Our observation of FA reduction in the genu of the CC is consistent with a previous meta-analysis of MDD that included VBA and TBSS studies^27^. However, other meta-analyses identified decreased FA values in the left superior longitudinal fasciculus, increased FA values in the right inferior fronto-occipital fasciculus^25^ and decreased FA values in the right frontal lobe, right fusiform gyrus, left frontal lobe and right occipital lobe^26^. These inconsistencies are attributable to distinct factors. First, the present meta-analysis only included DTI studies using TBSS and excluded studies using VBA, thus eliminating the potential for bias due to methodological differences in MRI data processing. Second, several studies that did not detect significant differences in FA between patients with MDD and HCS were included in the present study, whereas prior meta-analysis excluded these studies because the activation likelihood estimation (ALE) method cannot be used for the studies with no significant group differences^26^. Finally, the patient sample characteristics (e.g., age, gender, illness duration, age at onset, subtype and severity) of the included studies differ among the meta-analyses.

The CC is the largest WM tract connecting corresponding regions of the cerebral cortex in the two cerebral hemispheres to integrate the motor, sensory, and cognitive functions of the brain^43^. In neuroimaging studies, the abnormalities of the CC have been increasingly implicated in MDD^44^,^45^,^46^. Structural MRI revealed that the genu region of the CC is smaller in early onset adult MDD^47^. Selected ROI analyses of DTI have also demonstrated that adult patients with MDD have reduced FA in the genu of the CC^48^, suggesting decreased structural integrity of its related WM commissural fibres. In addition, magnetization transfer ratio (MTR) imaging has also revealed a lower MTR in the genu of the CC in patients with MDD compared with non-depressed controls^49^.

The WM fibres passing through the genu of the CC connect the bilateral prefrontal and orbitofrontal cortices, which are related to decision-making, attention, reward processing, and emotion regulation. Specific WM pathologies contributing to low FA could include myelin and/or axonal damage and gliosis^50^. The genu of the CC and prefrontal WM are both late-myelinating and are therefore more vulnerable to damage than the early myelinating splenium. The reduction of myelination in the genu of the CC could lead to decreased speed or quantity of interhemispheric communications^51^. The disturbed connectivity between these brain regions may negatively affect the normal process of interhemispheric information transfer in MDD patients, which may subsequently lead to deficits in memory, executive functioning and emotional regulation with predisposition toward more severe depressive symptoms^33^,^52^. Deficits in neuropsychological functioning can influence the daily functioning and worsen the quality of life of these patients. Our findings, together with those of previous studies, further support the assertion that MDD is associated with the disorganization of WM architecture in the genu of the CC, which connects prefrontal and orbitofrontal cortices in the two cerebral hemispheres.

Interestingly, the subgroup analysis of first-episode, treatment-naive datasets revealed no significant differences in FA in the genu of the CC, which indicated that the FA reduction in this area might be due to medication. Indeed, only one study in the included first-episode, treatment-naive datasets reported the FA reduction in the genu of the CC in patients with MDD. Another possible explanation of this negative finding in first episode cases is that FA reduction in the genu of the CC might be a “scar” caused by depressive illness duration. This speculation was supported by a previous DTI study, which demonstrated that illness duration of MDD was the predictor of whole-brain mean FA, with a significant negative linear relationship^53^. However, the results of subgroup analysis should be interpreted with caution because only 7 datasets of small sample size were recruited. Further investigations with longitudinal design are needed for a more comprehensive understanding of the WM abnormalities in different phases of MDD.

The ALIC is the WM tract between the head of the caudate nucleus and the lenticular nucleus, which is heavily involved in motivation, decision-making, and evaluating the saliency of emotional and rewarding stimuli^54^. The observed FA reduction in the ALIC implicates an impairment of the integrity of WM fibre tracts, which is particularly important in understanding the pathophysiology of MDD and is a useful addition to the findings obtained in the previous meta-analysis by Wise *et al.* (2015). Subgroup analysis of first-episode, treatment-naive MDD or medication-free MDD revealed that the FA reduction in the left ALIC remained unchanged. In the present meta-analysis, the fibre tract passing through the left ALIC with decreased FA, as identified by DTIquery software, was the anterior thalamic radiation connecting the medial dorsal thalamic nuclei with the prefrontal cortices, which is the main WM fibre tract passing from the ALIC and the large number of horizontally cut fibres in the ALIC^55^.

Disrupted or decreased demyelination or decreased fibre density or coherence in the region may be the biological mechanism underlying the decrease in FA in MDD. Abnormalities of the ALIC have been reported in patients with depression. A DTI study of adult depression using the VBA method revealed significantly decreased FA in the left ALIC^19^. Other DTI studies using the VBA method have also indicated decreased FA in the left ALIC in suicide attempters compared to both non attempters and HCS^56^ and reduced fibre projections through the ALIC to the left medial frontal cortex, orbitofrontal cortex and thalamus in depressed patients^57^. Furthermore, the ALIC is the most frequent therapeutic target for deep brain stimulation in treatment-resistant depression^58^,^59^. The FA reduction in the left ALIC may reflect a disconnection of frontal-striatal-thalamic neuronal circuits, which may cause damage to executive function and emotional lability in MDD patients^60^. Because the frontal-striatal-thalamic neuronal circuits consist of massive bundles of fibres passing through the ALIC, this disorganization of WM architecture in the ALIC further indicates an important role of frontal-striatal-thalamic pathways in MDD pathogenesis.

Meta-regression analysis demonstrated that the severity of depressive symptoms (HAMD scores) was negatively correlated with FA reduction in the genu of the CC, which indicates that the symptom severity of patients with MDD influences the degree of WM disruption to some extent. This finding is consistent with a previous DTI study that correlated greater depression severity with reduced WM integrity in the genu of the CC^54^. These converging lines of evidence further demonstrate that abnormalities in structural connectivity are associated with the pathophysiology of MDD. However, there was no significant correlation between FA reduction in the left ALIC and symptom severity, consistent with the findings of previous DTI studies that used the VBA method^56^. Conversely, another DTI study observed that FA reduction in the left ALIC is negatively related to the severity of depressive symptoms^19^. These discrepancies are likely due to variations of age, gender, illness duration, age at onset, subtype and medication status in patients with MDD.

The present study has several limitations. First, the number of DTI studies with TBSS in adolescent or late-life depression was small. Thus, we only included studies of adult depression and were not able to compare differences in FA in various age ranges. Second, the heterogeneity of the MRI data acquisition, including voxel size, diffusion direction, and slice thickness, may decrease the accuracy of the results of the present meta-analysis. Third, the included studies varied in terms of patient characteristics and clinical variables. For example, the patients with MDD included in the present study had either first-episode or recurrent MDD. Although we performed a subgroup meta-analysis for the first-episode, treatment-naive and medication-free datasets, the results should be interpreted with caution. There are only 7 first-episode, treatment-naive datasets, and the small sample size of the subgroup meta-analysis limits the generalizability of the results. These first-episode MDD patients could potentially experience manic or hypomanic episodes in the future and may be diagnosed with bipolar disorder. Compared with unipolar depression, bipolar disorder has more widespread abnormalities in WM connectivity and WM hyperintensities^61^. In addition, the washout period prior to MRI scanning varied among the included studies and was as short as only 2 days. Therefore, the subgroup meta-analysis of medication-free datasets may not completely exclude the effects of medication. Fourth, while it is true that TBSS appears to be a more sensitive method than VBA, omitting these studies using VBA reduces the number of the included studies and gives a somewhat incomplete picture of the literature. Finally, we only focused on FA and did not consider other measures of diffusivity, such as mean, radial or axial diffusivity, which may provide insights on the nature of the underlying WM changes contributing to alterations in FA.

In summary, this meta-analysis of DTI studies with TBSS identified two consistent locations of FA reduction in patients with MDD. The largest cluster, located in the genu of the CC and extending to the body of the CC, represents the interhemispheric fibres connecting the prefrontal and orbitofrontal cortices. The other cluster of FA reduction, located in the left ALIC, is the anterior thalamic radiation connecting the medial dorsal thalamic nuclei with the prefrontal cortices. These findings integrate previous inconsistencies in the DTI studies of MDD and provide a coherent picture of the most prominent and replicable abnormalities of WM in patients with MDD. Furthermore, our meta-regression analysis provides evidence suggesting that the symptom severity of patients with MDD is negatively associated with FA reduction in the genu of the CC. Our results demonstrate that the abnormalities of interhemispheric connections and frontal-striatal-thalamic pathways may play an important role in MDD pathogenesis.

## Methods

### Literature search strategy

Systematic and comprehensive searches of the PubMed, Web of Science, PsycINFO, Cochrane Library, and EMBASE databases were performed for studies published between January 1994 and July 2015 and “in press” articles. The search keywords were (“unipolar disorder” or “depressive disorder” or “depression”) and (“tract-based spatial statistics” or “TBSS”) and (“diffusion tensor” or “DTI”). The reference lists of the identified articles and review articles were also manually reviewed to identify additional papers.

### Study eligibility criteria

All DTI studies using a TBSS approach yielded by our search were assessed for potential suitability, and the articles that met the following inclusion criteria were adopted for the meta-analysis: (i) published in a peer-reviewed English language journal; (ii) included adult patients with MDD to minimize the influence of neurodevelopment and neurodegeneration on WM diffusion; (iii) compared a group of MDD patients according to the Diagnostic and Statistical Manual of Mental Disorders, Fourth Edition (DSM-IV) criteria with a group of HCS; (iv) utilized TBSS to investigate FA alterations between MDD patients and HCS; (v) used thresholds for significance corrected for multiple comparisons or uncorrected with spatial extent thresholds; and (vi) reported whole-brain three-dimensional coordinates (Talairach or Montreal Neurological Institute) of FA alterations in a stereotactic space to enable voxel-level quantitative meta-analysis. For publications that were otherwise suitable for meta-analysis but did not report whole-brain coordinates, the corresponding authors were contacted for additional information.

The studies were excluded if they had at least one of the following deficiencies: (i) the studies were case reports or reviews; (ii) the studies included adolescent patients with depression or patients with late-life depression; (iii) the three -dimensional coordinates in stereotactic space could not be obtained; (iv) the data were entered twice from a study population that had been analysed in more than one publication; (v) participants with multiple combined Axis I diagnoses were explicitly recruited; and (vi) fewer than ten subjects in either the MDD group or the HCS group were studied. The method used in the current study was in accordance with the Meta-analysis Of Observational Studies in Epidemiology (MOOSE) guidelines for meta-analyses of observational studies^62^.

### Quality assessment and data extraction

Two authors (G.X.C. and X.Y.H.) independently searched the literature, examined the retrieved articles, and extracted and cross-checked data. The quality of the included studies were also independently assessed by the two authors using a 12-point checklist (see the Supplementary Information, Table S1) that focused on both the clinical and demographic aspects of individual study samples and on the imaging-specific methodology^63^. If agreement was not obtained, one of the authors (L.L.) mediated. The quality scores for each study are provided in Table 1. For each included study, the following data were extracted: demographic information including participant characteristics (sample size, age, and gender), age at onset, illness duration, symptom severity, diagnosis, statistical threshold and drug status; the three-dimensional coordinates in each study were also extracted for the meta-analysis according to the AES-SDM method^64^, the main features of which are the possibility of combining peak coordinates and statistical parametric maps, as well as the use of well-established statistics accounting for within- and between-study variance^65^.

### Voxel-wise meta-analysis

Voxel-wise meta-analysis was performed on the selected studies using AES-SDM software in a standard process to compare FA changes between the MDD group and HCS group. Descriptive analysis of quartiles was also performed to check the actual proportion of the studies reporting results in a particular brain region. Systematic whole-brain voxel-based jack-knife sensitivity analysis was performed to test the replicability of the results. To control for any possible influence of drug status between studies, subgroup meta-analyses of the first-episode, treatment-naive and medication-free datasets were conducted. The AES-SDM software editor was also contacted via email when necessary. These analytical processes refer to the AES-SDM tutorial (http://sdmproject.com/software/Tutorial.pdf) and related publications^64^,^66^. The analytical parameters of the AES-SDM were as follows: anisotropy = 1.0; isotropic full-width at half-maximum (FWHM) = 20 mm; voxel *p* = 0.005; peak height threshold = 1; cluster extent = 10 voxels with 10 repetitions of standard randomization tests. Moreover, we used MRIcron software (http://www.cabiatl.com/mricro/mricron/) to visualize AES-SDM maps overlaid onto a high-resolution brain template generated by the International Consortium for Brain Mapping. DTIquery software (http://graphics.stanford.edu/projects/dti/) and an atlas of human WM anatomy^67^ was applied to help identify the fascicles involved in each region. We used the sample data of a healthy 35-year-old male provided by DTIquery software. The possible existence of publication bias for the brain regions with FA alterations was assessed by Egger’s test using Stata software (version12.0).

### Meta-regression analysis

The potential effects of the mean age, illness duration, symptom severity (HAMD scores) and drug status of the MDD participants were examined by simple linear regression, weighted by the square root of the sample size and restricted to predict only possible AES-SDM values (i.e., from −1 to 1) in the observed range of values of the variable. The main output for each variable was a map of the regression slope^30^. As described in previous meta-analyses, to minimize the detection of spurious relationships, we decreased the probability threshold to 0.0005 required abnormalities to be detected both in the slope and in one of the extremes of the regressor and discarded findings in regions other than those detected in the main analyses. Regression plots were visually inspected to discard fittings driven by too few studies^68^.

## Additional Information

**How to cite this article**: Chen, G. *et al.* Disorganization of white matter architecture in major depressive disorder: a meta-analysis of diffusion tensor imaging with tract-based spatial statistics. *Sci. Rep.* **6**, 21825; doi: 10.1038/srep21825 (2016).

## Supplementary Material

Supplementary Information

## Figures and Tables

**Table 1:** Summary of the 17 DTI studies (18 datasets) with TBSS included in the meta-analysis.

Study	Subjects, n (females, n)		Age, years		Age at onset years,mean	Illness duration, years	Severity (scale type)	Diagnosis	Statistical threshold	Drug status	Diffusion directions	Quality scores (out of 12)
	Patients	Controls	Patients	Controls								
Zuo *et al.* 2012 [20]	16 (13)	19 (12)	37.0 ± 9.4	36.6 ± 7.7	NA	NA	30.3 ± 6.2 (HAMD)	MDD	P < 0.005 (Uncorrected)	Medication free for >2 weeks	25	11
Lai *et al.* 2014 [21]	44 (23)	27 (15)	36.9 ± 5.3	38.3 ± 11.8	NA	0.4 ± 0.1	22.1 ± 2.3 (HAMD)	First-episode MDD	P < 0.05 (FWE)	drug naive	30	11
Zhu *et al.* 2011 [22]	25 (15)	25 (15)	20.6 ± 1.9	20.3 ± 1.7	20.2 ± 2.3	0.9 ± 0.7	35.5 ± 6.7 (CES-D)	First-episode MDD	P < 0.05 (corrected for multiple comparisons)	drug naive	13	10.5
Versace *et al.* 2010 [23]	16 (12)	24 (15)	32.9 ± 10.0	27.7 ± 8.6	18.9 ± 7.2	14.7 ± 10.0	25.1 ± 5.5 (HAMD)	Recurrent unipolar depression	P < 0.05 (ASC)	Medication free for >2 months	NA	11.5
Choi *et al.* 2014 [24]	134 (70)	54 (26)	38.5 ± 11.1	34.4 ± 10.1	NA	9.3 ± 10.4	19.3 ± 3.5 (HAMD)	MDD	P < 0.05 (FWE)	98 drug naive,36 medication free	60	12
Murphy *et al.* 2012 [31]	45 (29)	45 (28)	42.2 ± 10.8	36.5 ± 13.4	NA	14.6 ± 11.5	28.9 ± 6.4 (HAMD)	MDD	P < 0.05 (FWE)	15 medication free,15 on SSRIs,15 on DASs	61	12
Guo *et al.* 2012 [32]	23 (12)	19 (9)	27.4 ± 7.7	24.4 ± 4.2	NA	2.3 ± 3.0	24.5 ± 4.2 (HAMD)	Treatment-resistant depression	P < 0.01 (corrected for multiple comparisons)	Antidepressants	13	10.5
Han *et al.* 2014 [33]	20 (15)	22 (15)	42.7 ± 12.4	43.7 ± 12.3	NA	0.37 ± 0.1	19.1 ± 6.7 (HAMD)	First-episode MDD	P < 0.01 (uncorrected)	drug naive	20	11.5
Hayashi *et al.* 2014 [34]	30 (13)	30 (13)	44.0 ± 12.0	44.0 ± 13.0	NA	NA	≥14.0 (HAMD)	First-episode MDD	P < 0.05 (FWE)	drug naive	25	10
Kieseppa *et al.* 2010 [35]	16 (14)	20 (10)	48.4 ± 10.3	42.0 ± 11.6	NA	14.1 ± NA	26.3 ± 7.1 (BDI)	MDD	P < 0.05 (corrected for multiple comparisons)	13 on antidepressants,1 on risperidone,4 on benzodiazepines or zopiclone	12	10
Lyden *et al.* 2014 [36]	20 (12)	28 (15)	41.2 ± 10.3	39.4 ± 12.1	19.9 ± 11.2	21.6 ± 12.5	27.4 ± 4.5 (HAMD)	Recurrent MDD	P < 0.05 (corrected for multiple comparisons)	Medication free for >2 days	61	11.5
Olvet *et al.* nMDD 2014 [37]	39 (24)	46 (21)	37.1 ± 11.4	30.3 ± 9.3	NA	NA	18.7 ± 4.7 (HAMD)	MDD	P < 0.05 (FWE)	Medication free for >2 weeks	25	11.5
Olvet *et al*. sMDD 2014 [37]	13 (7)	39 (21)	33.4 ± 13.3	30.3 ± 9.3	NA	NA	19.9 ± 4.8 (HAMD)	MDD	P < 0.05 (FWE)	Medication free for >2 weeks	25	11.5
Seok *et al.* 2013 [38]	86 (68)	62 (41)	44.7 ± 12.2	42.1 ± 14.5	NA	3.6 ± 3.8	14.6 ± 8.1 (HAMD)	MDD	P < 0.01 (FWE)	45 on antidepressants,41 drug naive	20	11
Guo *et al.* 2012 [39]	22 (10)	19 (9)	28.1 ± 9.9	24.4 ± 4.2	NA	0.2 ± 0.1	25.9 ± 6.3 (HAMD)	First-episode MDD	P < 0.01 (corrected for multiple comparisons)	drug naive	13	10.5
Korgaonkar *et al*. 2011 [40]	29 (17)	39 (21)	40.5 ± 15.8	29.6 ± 12.7	NA	NA	19.1 ± 3.0 (HAMD)	MDD	P < 0.05 (FWE)	Medication free or drug naive	42	10.5
Wang *et al.* 2014 [41]	41 (20)	41 (20)	32.4 ± 6.5	32.6 ± 5.3	NA	NA	23.8 ± 6.1 (HAMD)	First-episode MDD	P < 0.001 (Uncorrected)	drug naive	12	11
Xiao *et al.* 2015 [42]	22 (12)	22 (12)	20.1 ± 1.6	20.8 ± 1.4	NA	NA	55.7 ± 5.8 (CES-D)	First-episode MDD	P < 0.001 (corrected for multiple comparisons)	drug naive	13	11

Abbreviations: DTI, diffusion tensor imaging; TBSS, tract-based spatial statistics; MDD, major depressive disorder; N.A., not available; FWE, family-wise error; HAMD, Hamilton Depression Rating Scale; nMDD, depressed patients without a history of suicide attempts; sMDD, depressed patients with a history of suicide attempts; ASC, AlphaSim correction; CES-D, Center for Epidemiological Studies Depression Scale; SSRIs: selective serotonin reuptake inhibitors; DASs: dual-acting substances.

**Table 2:** Clusters of FA reductions in patients with major depressive disorder compared to healthy control subjects.

Region	Maximum			Cluster	
	MNI coordinates x, y, z	AES-SDM value	P value	Number of voxels	Breakdown (number of voxels)
Genu of CC	−6, 26, 10	−0.112	∼0	499	Genu of CC (307) Body of CC (182)
Left ALIC	−14, 4, 8	−0.068	0.000098121	73	Left ALIC (49) Left PLIC (17)

Abbreviations: FA, fractional anisotropy; CC, corpus callosum; ALIC, anterior limb of internal capsule; PLIC, posterior limb of internal capsule; AES-SDM, anisotropic effect size-signed differential mapping; MNI, Montreal Neurological Institute.

**Table 3:** Results of the reliability and subgroup analysis of findings from the 17 DTI studies (18 datasets) with TBSS included in the meta-analysis.

Analysis	Region of FA reduction: Genu of CC	Region of FA reduction: ALIC
Jackknife sensitivity analysis (discarded study)		
Excluding Zuo *et al.* 2012 [20]	Yes	Yes
Excluding Lai *et al.* 2014 [21]	Yes	Yes
Excluding Zhu *et al.* 2011 [22]	Yes	Yes
Excluding Versace *et al.* 2010 [23]	Yes	Yes
Excluding Choi *et al.* 2014 [24]	Yes	Yes
Excluding Murphy *et al.* 2012 [31]	Yes	Yes
Excluding Guo *et al.* 2012 [32]	Yes	Yes
Excluding Han *et al.* 2014 [33]	Yes	Yes
Excluding Hayashi *et al.* 2014 [34]	Yes	Yes
Excluding Kieseppa *et al.* 2010 [35]	Yes	Yes
Excluding Lyden *et al.* 2014 [36]	Yes	Yes
Excluding Olvet *et al.* nMDD 2014 [37]	Yes	No
Excluding Olvet *et al.* sMDD 2014 [37]	Yes	Yes
Excluding Seok *et al.* 2013 [38]	Yes	Yes
Excluding Guo *et al.* 2012 [39]	Yes	No
Excluding Korgaonkar *et al.* 2011 [40]	Yes	Yes
Excluding Wang *et al.* 2014 [41]	Yes	Yes
Excluding Xiao *et al.* 2015 [42]	Yes	No
Descriptive analysis of quartiles		
First quartile (25%)	Yes	No
Second quartile (median)	Yes	No
Third quartile (75%)	No	No
Subgroup analysis		
Studies with first-episode, treatment-naive MDD (n = 7)	No	Yes
Studies with Medication free MDD (n = 14)	Yes	Yes

“Yes” indicates that the brain region with FA reduction remained significant in the jack-knife analysis, descriptive analysis of quartiles and subgroup analysis; “No” indicates that the brain region with FA reduction was no longer significant in those analyses.

Abbreviations: DTI, diffusion tensor imaging; TBSS, tract-based spatial statistics; FA, fractional anisotropy; CC, corpus callosum; ALIC, anterior limb of internal capsule; MDD, major depressive disorder; nMDD, depressed patients without a history of suicide attempts; sMDD, depressed patients with a history of suicide attempts.
