# Supplementary material for: Disorganization of white matter architecture in major depressive disorder: a meta-analysis of diffusion tensor imaging with tract-based spatial statistics
Source: Sci Rep. 2016 Feb 24;6:21825. doi: 10.1038/srep21825 (PMC4764827; doi:10.1038/srep21825)
Supplement: Supplementary Information [file srep21825-s1.doc]

**Disorganization of white matter architecture in major depressive disorder: a meta-analysis of diffusion tensor imaging with tract-based spatial statistics**

Guangxiang Chen^1,2＃^, Xinyu Hu^1＃^, Lei Li^1^, Xiaoqi Huang^1^, Su Lui^1^, Weihong Kuang^3^, Hua Ai^4^, Feng Bi^5^, Zhongwei Gu^4^ & Qiyong Gong^1,6＊^

Supplementary Table S1: Imaging Methodology Quality Assessment Checklist (When criteria were partially met, 0.5 points were assigned)

| **Category 1: Subjects**  **Score** (0/0.5/1) |
| --- |
| 1 Patients were evaluated prospectively, specific diagnostic criteria were applied, and demographic data was reported |
| 2 Healthy comparison subjects were evaluated prospectively, psychiatric and medical illnesses were excluded |
| 3 Important variables (e.g. age, gender, illness duration, onset time, medication status, comorbidity, severity of illness) were checked, either by stratification or statistically |
| 4 Sample size per group > 10 |
| **Category 2: Methods for image acquisition and analysis** |
| 5 Magnet strength at least 1.5T |
| 6 MRI slice-thickness≤3 mm |
| 7 Whole brain analysis was automated with no a-priori regional selection |
| 8 Coordinates reported in a standard space |
| 9 The imaging technique used was clearly described so that it could be reproduced |
| 10 Measurements were clearly described so that they could be reproduced |
| **Category 3: Results and conclusions** |
| 11 Statistical parameters for significant, and important non-significant, differences were provided |
| 12 Conclusions were consistent with the results obtained and the limitations were discussed |
| **TOTAL**  /12 |
